# Breath-Jockey: Development and Feasibility Assessment of a Wearable System for Respiratory Rate and Kinematic Parameter Estimation for Gallop Athletes

**DOI:** 10.3390/s21010152

**Published:** 2020-12-29

**Authors:** Joshua Di Tocco, Riccardo Sabbadini, Luigi Raiano, Federica Fani, Simone Ripani, Emiliano Schena, Domenico Formica, Carlo Massaroni

**Affiliations:** 1 Unit of Measurements and Biomedical Instrumentation, Università Campus Bio-Medico di Roma, Via Alvaro del Portillo, 00128 Rome, Italy; j.ditocco@unicampus.it (J.D.T.); r.sabbadini@unicampus.it (R.S.); e.schena@unicampus.it (E.S.); 2 Unit of Neurophysiology and Neuroengineering of Human Technology Interaction (NeXT), Università Campus Bio-Medico di Roma, Via Alvaro del Portillo, 00128 Rome, Italy; l.raiano@unicampus.it (L.R.); d.formica@unicampus.it (D.F.); 3 Avery Dennison RBIS Italy, Prov.le Bonifica, 64010 Ancarano, Italy; federica.fani@eu.averydennison.com (F.F.); simone.ripani@eu.averydennison.com (S.R.)

**Keywords:** sensors, biomedical monitoring, monitoring, smart textiles, equine sport, clothing, instruments, jockey, horse racing, physiological parameters

## Abstract

In recent years, wearable devices for physiological parameter monitoring in sports and physical activities have been gaining momentum. In particular, some studies have focused their attention on using available commercial monitoring systems mainly on horses during training sessions or competitions. Only a few studies have focused on the jockey’s physiological and kinematic parameters. Although at a glance, it seems jockeys do not make a lot of effort during riding, it is quite the opposite. Indeed, especially during competitions, they profuse a short but high intensity effort. To this extend, we propose a wearable system integrating conductive textiles and an M-IMU to simultaneously monitor the respiratory rate (RR) and kinematic parameters of the riding activity. Firstly, we tested the developed wearable system on a healthy volunteer mimicking the typical riding movements of jockeys and compared the performances with a reference instrument. Lastly, we tested the system on two gallop jockeys during the “$137^{\circ }$ Derby Italiano di Galoppo”. The proposed system is able to track both the RR and the kinematic parameters during the various phases of the competition both at rest and during the race.

## 1. Introduction

Professional flat horse racing is a very challenging and demanding sport not only due to the high number of training hours, but also due to the effort jockeys make during the race day [1,2].

In this arena, while many investigations have been made on jockeys’ injuries [3,4,5] and horse vital parameters [6,7], only few studies have focused on monitoring the physiological and kinematic parameters of jockeys. In [8], the authors assessed heart rate (HR), oxygen uptake ($\dot{V}O_{2}$) and respiratory rate (RR) using commercial devices (i.e., Cosmed K4b2, Polar, and Equivital EQ02) in simulated and competitive race scenarios. As a result, the authors provided quantitative indexes for the physiological demand of jockeys during races confirming how they have to be fit to achieve a high quality of performance. In [2,9] the authors assessed the physiological demand of jockeys using a different approach. They measured the HR and assessed the effort made by jockeys by measuring the lactate concentration underlining how this sport is demanding and is testified by the HR fluctuations according to the race distances. In [10,11,12], the authors presented global positioning systems to estimate both the riding posture of the jockeys and some kinematic parameters of the horse during training or racing (e.g., number of strides and speed). The authors highlighted how a jockey interacts with horse movement during riding. Moreover, horse speed information during races is approximately 60 km·h${}^{-1}$ which emphasises why the focus has been made on injuries caused by a horse fall. Simultaneous monitoring of kinematic and physiological parameters may be beneficial to get insights into the relationship between physical stress and jockey effort. The lack of studies focusing on this topic is a major weakness of the literature. This lack can be filled using different technologies which are broadly accepted for the development of wearable devices devoted to monitor athletes in different scenarios (e.g., running, jogging, and precision sports) [13,14,15,16,17,18]. Among others, conductive textiles provide an optimal solution for developing wearable systems due to their high integrability and cost effectiveness. Moreover, their use combined with Magneto-Inertial Measurement Units (M-IMU) allows monitoring both kinematic and vital signs related to effort (e.g., RR) in challenging scenarios due to the presence of breathing-unrelated movements [16]. RR is gaining broad acceptance because it can provide fundamental information due to its sensitivity to psychological and physiological stressors [19,20,21]. In particular, [22,23] have shown how RR must be taken into account for high intensity dynamic tasks in a short time window. High values of RR are an indicator of effort and, unlike the most common fatigue markers (i.e., HR, Blood Lactate and VO2), it varies rapidly almost instantaneously as the intensity of effort varies.

The aim of this study is to develop and test a custom wearable device to monitor jockeys’ RR and the kinematic parameters (i.e., linear accelerations) associated to the acceleration the jockey undergoes during riding. We developed and tested conductive sensing elements to estimate their metrological properties and to design an adequate front-end electronics. Then, we developed a wearable system with the mentioned sensors and an M-IMU module. Finally, we assessed the feasibility of the wearable system on a healthy volunteer mimicking a real scenario and we tested the system on two jockeys in the “$137^{\circ }$ Derby Italiano di Galoppo”.

## 2. Characterization and Development of the Wearable System

In this section, we describe the sensing elements (i.e., conductive textiles) and their metrological characterization, as well as how the wearable system has been instrumented with the abovementioned sensing elements and the M-IMU.

### 2.1. Metrological Characterization of Sensing Elements for Respiratory Rate Monitoring

**Figure 1:**
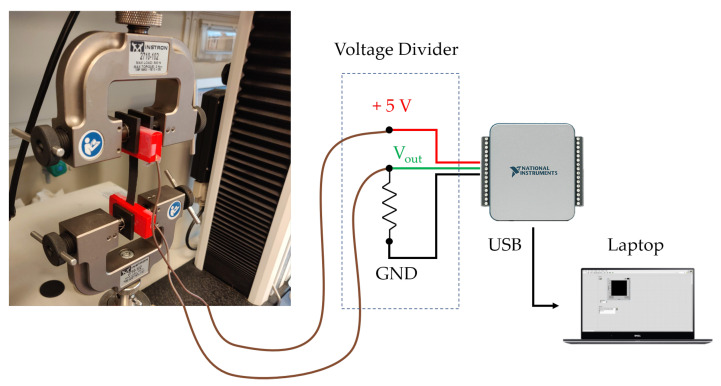
Experimental setup used for the characterization of the sensing elements.

The sensing elements used for RR monitoring have been characterized under both static conditions and during hysteresis cycles. This deep investigation was needed since we developed elements with custom shapes and sizes. The sensing element is a conductive fabric (EeonTeXTM LG-SLPA by Eeonyx, Pinole, CA, United States) about 0.6 mm thick cut in a 50 mm × 10 mm (length × width) rectangular shape. When this fabric undergoes strain (ϵ), its electrical resistance (R) changes. The amount of resistance change (ΔR) is related to the applied strain. To assess the metrological properties of the sensing element and to evaluate its use for respiratory monitoring, static and hysteresis tests were performed using a traction/compression testing machine (Instron 3365 by Illinois Tool Works Inc, Norwood, MA, United States). The sensor was positioned between the machine clamps and connected via two copper wires to a voltage divider allowing transducing the ΔR due to strain into voltage variations. An acquisition board (NIDAQ 6002 by National Instruments, Austin, TX, United States) was used both to power the voltage divider at +5 V and to record its output, see Figure 1.

#### 2.1.1. Static Analysis: Calibration Curve and Sensitivity Analysis

**Figure 2:**
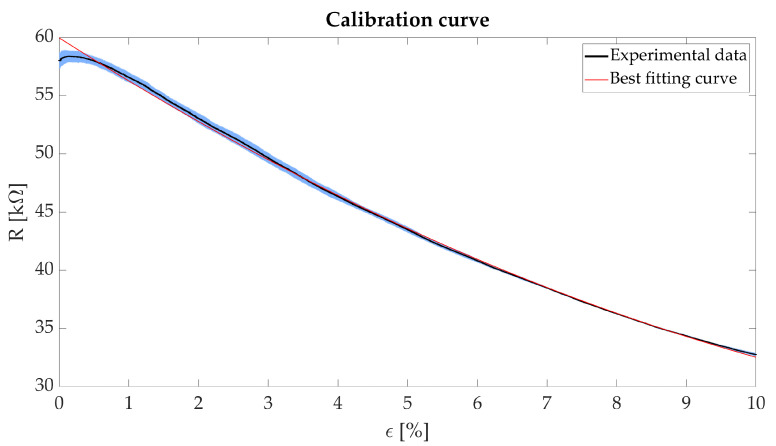
Plot showing the R vs. ϵ (black line), its related expanded uncertainty (blue shaded area) and the calculated calibration curve (red line).

We performed four static tests by setting two parameters: (i) the speed to strain the sensing element (a low speed of 1.5 mm·min${}^{-1}$ was set, to mimic quasi-static condition) and (ii) the maximum strain applied to the sensing element (i.e., $\epsilon_{max}$ = 10% was set). This $\epsilon_{max}$ value comprises respiratory-induced deformations of the rib cage which the sensing element will undergo for this specific application [24]. The ΔR of the sensing element due to the applied ϵ have been calculated by the voltage divider equation. Figure 2 shows R plotted versus ϵ.

To calculate the calibration curve, we recorded both the sensing element output (R) and input (ϵ). The acquired data were elaborated in a MATLAB^®^ environment using a custom algorithm. The average ΔR and its related uncertainty was calculated from the four performed trials. The calibration curve has been calculated as the best fitting quadratic polynomial of the experimental data. Figure 2 shows the obtained results reported as mean ± expanded uncertainty. The uncertainty has been calculated according to [25].

The agreement between the polynomial fit and the experimental data is confirmed by the high value of the correlation coefficient (R${}^{2}$ > 0.99). For ϵ ranging from 1% to 10%, there is both a better fit of the experimental data compared to the range 0–1% and a higher sensitivity. Therefore, the sensing element will be embedded in the wearable system using a pre-strain of approximately 1%. This solution will allow the sensing element to work in the range of values showing the best sensitivity. We calculated the mean sensitivity ($S_{mean}$) in this operating range (i.e., $\epsilon_{min}$ = 1%, $\epsilon_{max}$ = 10%) as in the following equation:

(1) $$S_{mean}=\frac{R\left(\epsilon_{max}\right)-R\left(\epsilon_{min}\right)}{\epsilon_{max}-\epsilon_{min}}$$

obtaining an $S_{mean}$ value of −2.65 k$\Omega \cdot \epsilon^{-1}$ in the range 1–10%. Its negative value is due to the decrease R with the increase of ϵ.

#### 2.1.2. Hysteresis Analysis

**Figure 3:**
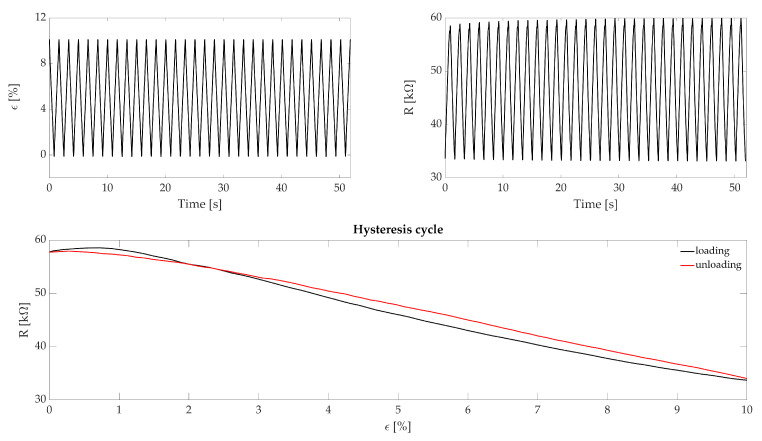
The top image represents the cyclic displacements provided to the sensor by the testing machine and the according ΔR. The bottom image represents a single hysteresis cycle where the black line represents the loading part and the red line the unloading of the cycle.

The hysteresis has been tested with 32 consecutive traction/compression cycles at four different speeds: (i) 2 mm·s${}^{-1}$ simulating an RR equal to 12 breaths per minute (hereinafter this measurement unit is indicated with the symbol bpm), (ii) 4 mm·s${}^{-1}$ simulating a RR equal to 24 bpm, (iii) 6 mm·s${}^{-1}$ simulating a RR equal to 36 bpm and (iv) 10 mm·s${}^{-1}$ simulating a RR equal to 60 bpm. Such RR values have been used to cover eupnea (quiet breathing with 12 bpm < RR < 21 bpm) and tachypnea (fast breathing with RR > 21 bpm) breathing regimes. Each cycle started from ϵ = 0% (i.e., initial length of the sensor), reached ϵ = 10% (i.e., maximum strain applied) and then terminated at ϵ = 0%. The first and last cycles have been used to synchronize the two recorded signals. Figure 3, shows the displacement and resistance change trends along with a single hysteresis cycle. To assess the hysteresis of the sensing element, we calculated the maximum percentage error ($err{\%}_{max}$) by dividing the maximum difference of ΔR between the ascending and the descending part of the hysteresis cycle at the same deformation value (indicated with $U_{0}$) by the maximum ΔR value ($r_{0}$), for each hysteresis cycle as in the following formula:

(2) $$err{\%}_{max}=\frac{U_{0}}{r_{0}}\cdot 100$$

The hysteresis analysis showed maximum errors of 3.6%, 5.0%, 5.4% and 7.0% corresponding to 12 bpm, 24 bpm, 36 bpm, and 60 bpm, respectively.

### 2.2. Sensing Unit for Monitoring Kinematic Parameters

A commercial Magneto-Inertial Measurement Unit, M-IMU (LSM9DS1 by STMicroelectronics, Geneva, Switzerland) was used to monitor the accelerations which the jokey undergoes. The above-mentioned unit uses an I2C protocol to collect data and allows monitoring the acceleration within a wide range of measurements (i.e., ±156.91 m·s${}^{-2}$). It is worth noting that this module suites the application due to its small size (i.e., 3.5 mm × 3 mm × 1.0 mm) and weight (∼22 mg).

### 2.3. Development of the Wearable System

The wearable system is a T-shirt embedding four sensing elements to detect the respiratory activity and a custom developed printed circuit board (PCB) for signal conditioning and data storage.

**Figure 4:**
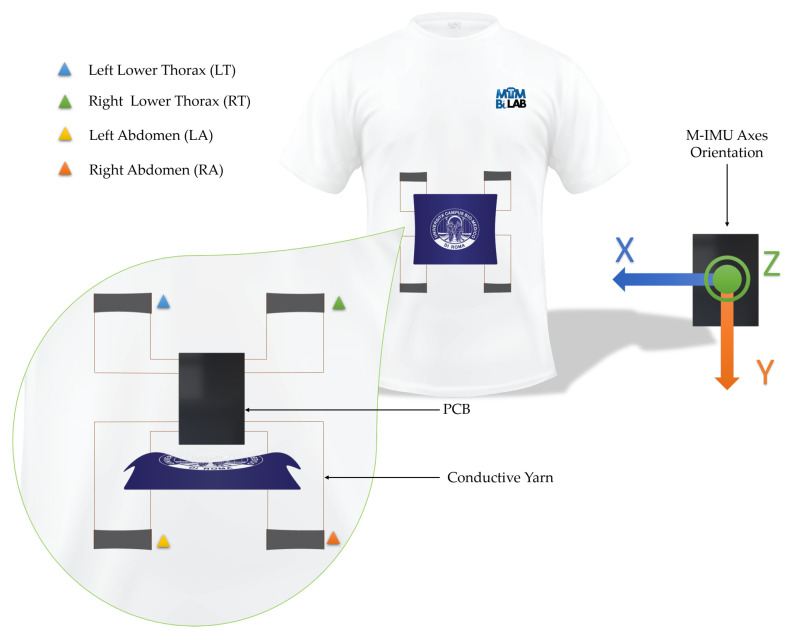
Graphical representation of the wearable device embedding the four sensing elements, the PCB used to collect the signals and the M-IMU axes.

The four elements were positioned as two on the lower thorax and two on the abdomen, as shown in Figure 4.

The sensing elements were sewed on a commercial T-shirt by means of a conductive yarn. The board is attached to the T-shirt through a polymeric adhesive (Euiooctory by VKAR). To limit the movements of the PCB during physical activity, it was inserted inside a custom pocket sewn on the chest. The positioning on the chest was chosen to minimize effects on jockeys during the race.

**Figure 5:**
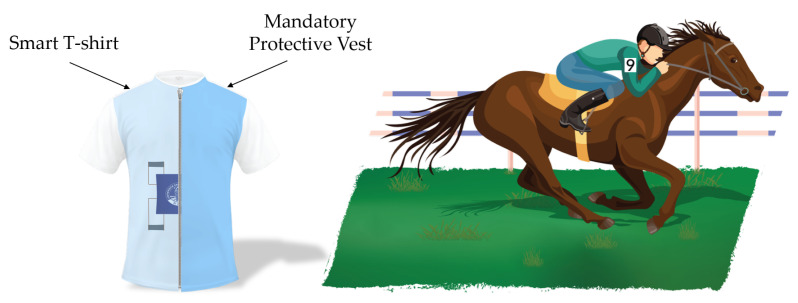
Schematic representation of how the wearable system is worn during the race (underneath the protective vest).

This annoyance is due to the position taken by the jockey during the race; in fact, the pose leads him to close in a crouched position [12] as shown in Figure 5.

The sensing elements capture the expansions and contractions of the rib cage and abdomen, respectively, during inhalation and exhalation.

The sensors are connected to the PCB through conductive wires sewn on the T-shirt. Four Wheatstone bridges convert the ΔR into a voltage variation which is amplified by the two on-board instrumentation amplifiers (AD8426 by Analog Devices, Norwood, MA, United States) and digitized by the analog-pdigital converter (ADC, MAX 1237 by Maxim Integrated, San Jose, CA, United States). The microcontroller (STM32F446RE by STMicroelectronics, Geneva, Switzerland) receives the digital signals from both the ADC and the M-IMU. The M-IMU is installed on the PCB itself and stores the data into a micro SD mounted inside the embedded SD-card socket.

## 3. Experimental Setup and Result

The proposed system was assessed in two different scenarios: (i) firstly, we performed a pilot test on a healthy volunteer mimicking the movements of jockeys during horse riding to investigate the capability of the system to estimate RR; (ii) then, we performed a feasibility assessment by using the wearable on two professional gallop athletes.

### 3.1. Test on a Healthy Volunteer

To asses the feasibility of the wearable system for respiratory monitoring, we asked a healthy volunteer to wear the T-shirt and to perform two tasks mimicking the respiratory and physical activity of a jockey before and during riding. In the first task, the volunteer was asked to breathe at self pace; the second simulated the up–down oscillating movements of the jockeys while self paced breathing. In both trials, a reference flowmeter was used to collect a reference value of RR with a system that can be considered as the gold standard. The outputs of the four sensing elements were filtered using a 3rd order Butterworth band-pass filter with high cut-off frequency of 1.5 Hz and low cutoff frequency of 0.05 Hz. This frequency range widely covers both eupnea and tachypnea breathing regimes [23,26].

**Figure 6:**
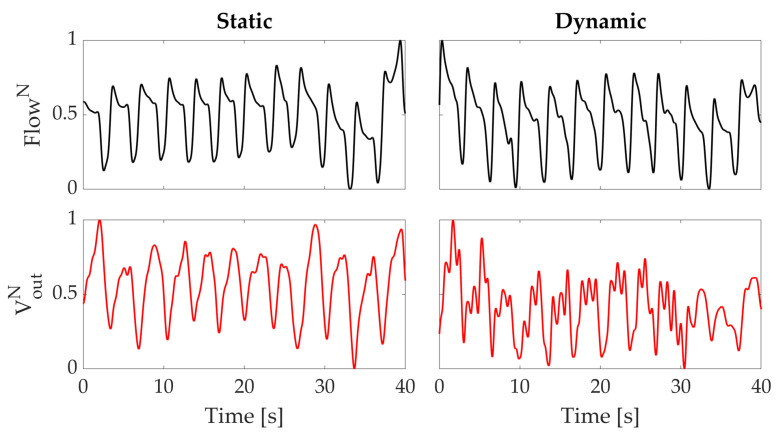
Normalized output data from the wearable system ($V_{out}^{N}$) and reference flowmeter ($Flow^{N}$) used. The left plots show the signals obtained in the static task; the right ones show the signals obtained in the dynamic task.

The data collected during these sets of experiments show that the proposed wearable system is able to follow the respiratory activity of the subject in both static and dynamic conditions. The normalized signals recorded by the flowmeter and by one of the sensing elements of the wearable system are shown in Figure 6. During the second task, the output of the sensing element is affected by the motion artefacts, but the identification of each breath can be achieved considering the minima of the signals (see Figure 6).

Indeed, comparing the data collected by the proposed wearable system with those of the reference instrument we found that both identify the same number of breaths and the associated RR in both static and dynamic conditions. In static conditions, we obtained 17.6 bpm for the reference instrument and 17.8 bpm for the wearable system; in dynamic conditions, 17.7 bpm and 17.6 bpm resulted for the reference instrument and wearable system, respectively. Therefore, the error on RR estimation, calculated as the difference between the RR estimated by the reference instrument and the proposed system, was −0.2 bpm and +0.1 bpm in static and dynamic conditions, respectively. Moreover, the associated average RR was determined by identifying each respiratory peak and calculating the reciprocal of the average time difference between consecutive peaks.

The proposed wearable system records along with the respiratory data also the 3-axis inertial data allowing the evaluation of the acceleration which the subject undergoes. Therefore, We calculated such acceleration as the Euclidean norm as in the following equation:

(3) $$a\left(t\right)=\sqrt{a_{x}^{2}\left(t\right)+a_{y}^{2}\left(t\right)+a_{z}^{2}\left(t\right)}$$

The maximum and average values of acceleration obtained were ∼14.2 m·s${}^{-2}$ and ∼9.7 m·s${}^{-2}$ respectively.

### 3.2. Pilot Study on Race Day

Two professional gallop athletes, one male and one female, were invited to wear the wearable system during the international competition “$137^{\circ }$ Derby Italiano di Galoppo”, which is the most important event of the competitive gallop season in Italy.

The anthropometric characteristics of the athletes are reported in Table 1.

Subjects wore the wearable system during the official dressing process before the overall weighing. In this way, they were able to verify the unobtrusiveness of our system. The overall weight of the wearable system was ∼200 g and therefore had no obstacle to overcome the limitations imposed by the competition regulations.

The jockeys wore the system underneath the mandatory protective vest as shown in Figure 5. The protective vest is slightly compressive, but the pressure exerted on the jockey’s chest is irisory. In addition, the vest helps to stabilize the board on the chest, effectively eliminating excessive vibrations of the case, which could drive the measuring system.

The principles of the Declaration of Helsinki were followed in all steps of the study and written informed consent for study participation was obtained from all the volunteers.

The monitoring started at the moment of dressing (i.e., between 30 and 50 min before the race beginning) until the moment of undressing the jockey (i.e., between 15 and 20 min after the race ended). The timestamps at which certain events occurred have been recorded; that is: (i) acquisition start; (ii) horse mounting; (iii) start of the race; (iv) end of the race; (v) horse dismounting; (vi) undressing; (vii) acquisition end. Starting from the knowledge of these seven events, three remarkable timestamps were identified:

1. Pre-race phase (∼1000 s before the race start)
2. Race phase
3. Post-race phase (∼1000 s after the race end)

**Figure 7:**
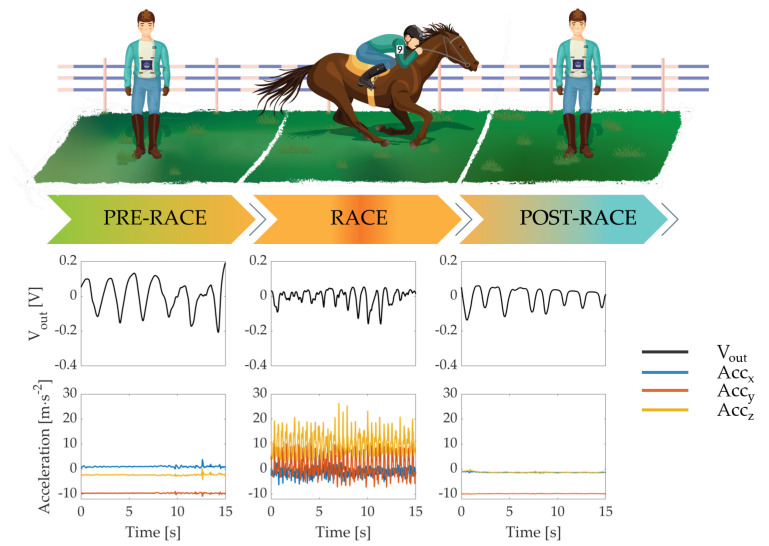
Graphical representation of the race phases and the related respiratory signal (black line) and the accelerometer data (blue, red and yellow lines).

Figure 7 shows both a graphical representation of the race phases identified and the retrieved respiratory signal ($V_{out}$) and the inertial data ($Acc_{x}$, $Acc_{y}$, $Acc_{z}$). It is worth noting that the amplitudes of the wearable system’s output reported in Figure 6 and Figure 7 are different because only in the first case the signals have been normalized. Instead, referring to Figure 7, the amplitude changes during the different race phases can be explained by position shift of the sensors on the chest wall caused by breathing movements.

The data were processed offline in MATLAB^®^ environment using a custom algorithm. Firstly, we identified three phases in the acquired signal by considering the time windows corresponding to the different race phases collected during race day. As a pre-processing, we filtered the respiratory data using a 3rd order band pass butterworth filter with high cutoff of 1.5 Hz and low cutoff frequency of 0.05 Hz and the M-IMU data using a 3rd order band pass butterworth filter with high cut-off of 5 Hz and low cutoff frequency of 0.05 Hz. Then, we performed the analysis on the data collected by the conductive sensing elements and the M-IMU.

We identified time windows up to 20 s in each race phase and retrieved the number of breaths by selecting the inspiratory peaks from the respiratory signal. Then, we obtained the average respiratory frequency ($RR_{avg}$) for each time window by calculating the reciprocal of the average time difference (Δt) between each consecutive breath.The $RR_{avg}$ values estimated during the three phases are shown in Table 2.

We identified the race phase time window and retrieved different race parameters:

(i) *Race duration*: time window calculated as the time difference between the end and the beginning of the race. The race duration for both jockeys was approximately 120 s.
(ii) *Acceleration*: the proposed wearable system records along with the respiratory data also the three-axis inertial data allowing the evaluation of the acceleration which the jockey undergoes. We calculated such acceleration as in Equation (3) The maximum and average values of acceleration were ∼36.3 m·s${}^{-2}$ and ∼9.8 m·s${}^{-2}$ for jockey 1 and ∼47.1 m·s${}^{-2}$ and ∼10.8 m·s${}^{-2}$ for jockey 2, respectively.
(iii) *Number of strides*: the number of horse strides has been calculated by summing the number of peaks in the jockey’s acceleration signal during the race phase, obtaining 293 strides for jockey 1 and 326 strides for jockey 2.
(iv) *Average horse stride length*: we calculated the average horse stride length by dividing the total length of the race circuit (i.e., 2000 m for jockey 1 and 2400 m for jockey 2) by the number of strides calculated previously obtaining a stride length of 6.83 m and 7.36 m, respectively.

## 4. Discussion and Conclusions

This paper introduces the development of a multisensor wearable system for monitoring RR and kinematic parameters in jockeys. Firstly, we assessed the sensing elements and the proposed system in a laboratory and on a healthy volunteer. Then, we performed for the first time the monitoring of RR and some kinematic parameters (i.e., linear acceleration and angular velocity) on two jockeys during an international gallop event. Indeed, many studies of wearable devices have been limited to investigations in restful conditions [27,28]; only in some cases, experiments were performed during physical activity [14,16], and investigations on jockeys and their physiological status are few and far between [2,8,9,10,11,12].

In this arena, previous studies used commercial systems, although tailored platforms can improve the data quality, as well as the comfortability and usability of the system [2,8,9]. The proposed system allowed collecting a vital sign (i.e., RR), as well as the linear acceleration reached during the race that are related to the jockey’s effort. The estimated RR showed how the respiratory activity becomes more intense during the race, underlining the effort made by the jockeys. Indeed, the M-IMU data showed that the jockeys underwent intense accelerations (i.e., the maximum value was approximately 47.1 m·s${}^{-2}$). Comparing the acceleration data of the race day with those from the simulation, the average values of acceleration are comparable and the maxima are not due to the absence of the horse which provides an additional contribute to the overall acceleration. The field tests, carried out in the context of a high-level event (“$137^{\circ }$ Derby italiano di galoppo”), showed that there is a significant increase in RR during the race. Indeed, RR increased between pre-race and race of approximately 14.5 bpm and 22.2 bpm for the two jockeys. Moreover, after the end of the athletic performance, the RR dropped to 8.8 bpm and 34.4 bpm for the two jockeys. During these tests, a reference system was not used, but these results are supported by the good performance shown by the proposed system on a volunteer mimicking jockey movements. The results were promising for both RR and kinematic parameters, indeed, the system seems to be able to capture the RR both at rest and during the race despite the breathing-unrelated movements due to a gallop.

Future tests will be focused on understanding the behaviour of the RR changes during the race by increasing the sample size; in addition, we will test the system on jockeys with different mount styles and in different race types, since these two conditions can affect the amplitude of the movements. In conclusion, in this work we have assessed the feasibility of a multisensor wearable system based on four conductive textiles and an M-IMU to detect both RR and kinematic parameters of professional athletes during a gallop race. This is the first study simultaneously monitoring these data on jockeys during a race.

## Figures and Tables

**Table 1:** Anthropometric characteristics declared by the volunteers.

Jockey	Height [cm]	Weight [kg]	BMI [kg/m${}^{2}$]	Sex	Age [y]
1	160	50	19.5	Male	26
2	158	48	19.2	Female	26

**Table 2:** Respiratory rate estimation in the different race phases.

Jockey	Pre-Race [bpm]	Race [bpm]	Post-Race [bpm]
1	24.0	38.5	29.7
2	30.1	52.3	17.8

## Data Availability

The data presented in this study are available on request from the corresponding author. The data are not publicly available due to privacy reason.

## Abbreviations

The following abbreviations are used in this manuscript:

RR	Respiratory Rate
M-IMU	Magneto Inertial Measurement Unit
HR	Heart Rate
PSD	Power Spectral Density
$\dot{V}O_{2}$	Oxygen uptake
I2C	Inter-Integrated Circuit
PCB	Printed Circuit Board
ADC	Analog Digital Converter
